# Supplementary material for: Asparaginase-Phage P22 Nanoreactors: Toward a Biobetter Development for Acute Lymphoblastic Leukemia Treatment
Source: Pharmaceutics. 2021 Apr 22;13(5):604. doi: 10.3390/pharmaceutics13050604 (PMC8170886; doi:10.3390/pharmaceutics13050604)
Supplement: Supplementary file 1 [file pharmaceutics-13-00604-s001.zip › pharmaceutics-1128361-supplementary.pdf]

pharmaceutics

Supplementary Material: Asparaginase-Phage P22 Nanoreactors: Toward a Biobetter Development for Acute Lymphoblastic Leukemia Treatment

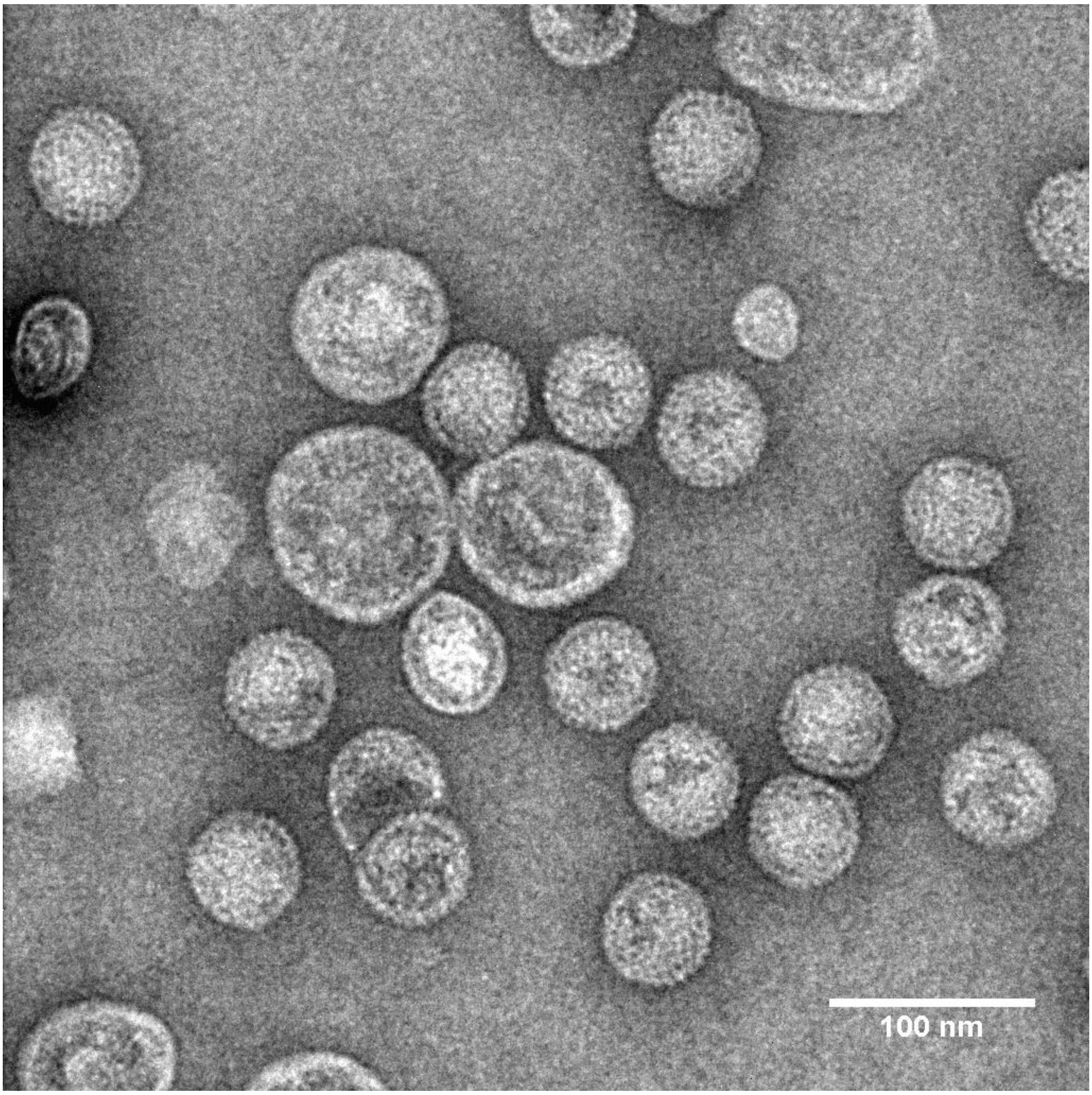

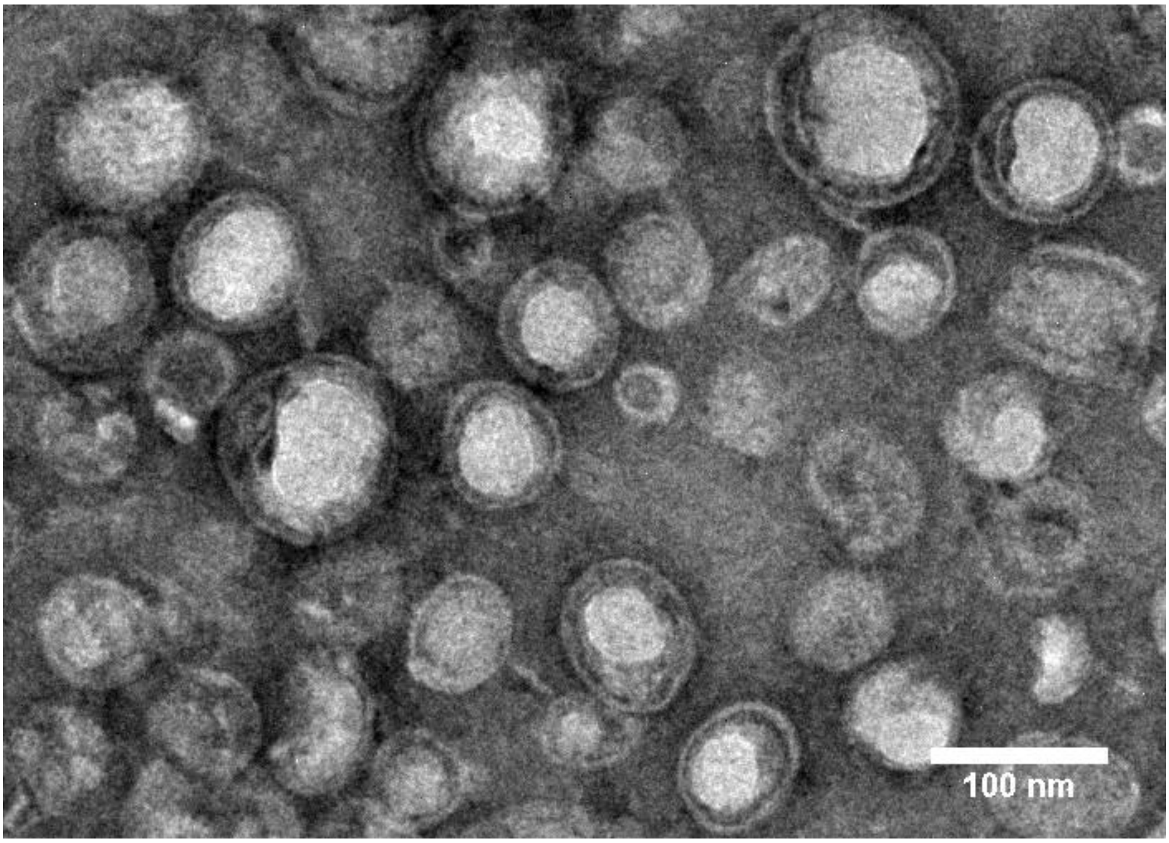

Figure S1. Additional TEM images of ASNase-P22 nanoreactors.

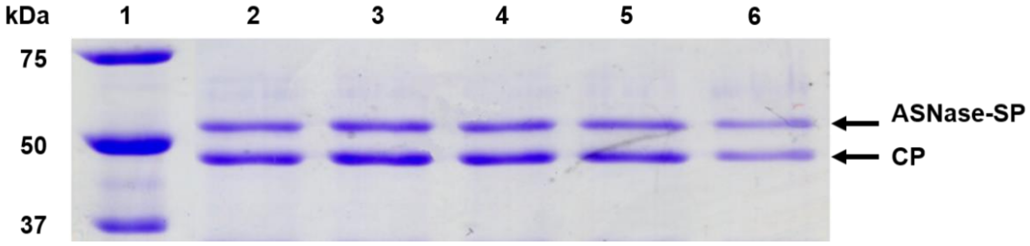

Figure S2. SDS-PAGE of purified samples of ASNase-P22 nanoreactors. Image used for densitometric analysis. Lane 1, molecular weight marker. Lanes 2-6 representative samples of ASNase-P22 from different chromatograms. Black arrows point out the ASNase-SP and CP bands at 53 kDa and 47 kDa, respectively.
